# Association study between common variations in some candidate genes and prostate adenocarcinoma predisposition through multi-stage approach in Iranian population

**DOI:** 10.1186/s12881-020-01014-0

**Published:** 2020-04-15

**Authors:** Behnaz Beikzadeh, Seyed Abdolhamid Angaji, Maryam Abolhasani

**Affiliations:** 1 Department of Cell and Molecular Biology, Faculty of Biological Sciences, Kharazmi University, Tehran, Iran; 2 Department of Pathology, School of Medicine, Iran University of Medical Sciences, Tehran, Iran

**Keywords:** Benign prostatic hyperplasia (BPH), PSA, 8q24, EHBP1, Single nucleotide polymorphism (SNP), KLK3

## Abstract

**Background:**

Prostate cancer is one of the five common cancers and has the second incidence rate and the third mortality rate in Iranian population. The purpose of this study was to evaluate the association of rs16901979, rs4242382 and rs1447295 on 8q24 locus, rs2735839 (KLK3 gene) and rs721048 (EHBP1 gene) with prostate adenocarcinoma through multi-stage approach to identify the polymorphisms associated with prostate cancer and use them as screening factors. Screening tests can identify people who may have a chance of developing the disease before detection and any symptoms.

**Methods:**

The case-control study included 103 cases (prostate adenocarcinoma) and 100 controls (benign prostatic hyperplasia). Tetra-primer ARMS-PCR was used to genotyping of each participant. A Multi-stage approach was used for efficient genomic study. In this method, a smaller number of people can be used. Chi-squared, Fisher’s exact test and logistic regression were used to investigate the SNPs associated with prostate cancer and Gleason score.

**Results:**

In the first stage (59 men), the frequency of polymorphisms rs16901979, rs4242382, rs1447295, rs2735839 and rs721048 in the prostate adenocarcinoma group was evaluated compared to the control group (*P*-value < 0.3) in order to select meaningful polymorphisms. There was not any significant difference between genotype frequency rs16901979 (*P* = 0.671) and rs721048 (*P* = 0.474) in the case group compared to BPH. Therefore, these polymorphisms were eliminated, and in the second step (144 men), rs4242382, rs2735839 and rs1447295 were evaluated (*P-value* < 0.05). According to the total population (203 men), there was significant difference between genotype frequency rs4242382 (*P* = 0.001), rs2735839 (*P* = 0.000) and rs1447295 (*P* = 0.005) even after using Bonferroni correction (*p* = 0.016). The effect of these three polymorphisms on prostate cancer was not modified by age and PSA. There was a significant difference between the allelic frequency of A vs G (rs4242382, rs2735839) at all classes of Gleason score and A vs C (rs1447295) at Gleason score ≥ 8.

**Conclusions:**

The results of this study for rs2735839, rs4242382 and rs1447295 indicate the association of these polymorphisms with prostate adenocarcinoma predisposition in Iranian population. Exposure effect is homogeneous between different ages and PSA level categories. These three polymorphisms should be studied in a larger population to confirm these results.

## Background

Prostate cancer is a malignancy, which in most cases is prostatic adenocarcinoma amongst men, in which tumor cells begin to grow and proliferation of the epithelial tissue [1–4]. About 70% of the prostate tumors originate from the peripheral zone, 25% from the Transition zone and 5% from the central zone [5–8]. The incidence of this cancer varies among different populations. The highest and the lowest prevalence have been found in the African-American and South Asian races respectively [9, 10]. Despite the trend toward declining in the incidence and mortality rates of prostate cancer in the United States and some other Western countries, the incidence of this cancer is increasing in less developed and developing country [9–12]. In Iran, the prevalence as with other developing countries, is increasing [13]. According to recent GLOBOCAN reports in 2018, Age-Standardized Incidence Rate (ASIR) and Age-Standardized Mortality Rate (ASMR) of prostate cancer were 16.6 and 8.3 per 100,000 populations in Iran. Therefore, it has the second incidence rate and the third mortality rate [13].

In the early stages of the cancer, when the tumor is limited to the prostate tissue itself, its symptoms are rare. But the main symptoms that arise when the cancer progresses include frequent urination, urinary incontinence, hematuria (blood in the urine), persistent pain in the lower back or pelvic cavity. Early diagnosis and accurate classification of prostate cancer are vital because survival rate is much reduced when the cancer spreads beyond the prostate. Because of tissue heterogeneity in prostate cancer, there is no specific imaging for the early diagnosis. Hence, the first step in diagnosis is usually the Prostate specific antigen (PSA) test and digital rectal exam (DRE) [2–4]. The PSA test, although is dedicated to the prostate gland, it is not proprietary for prostate cancer [14]. Benign prostatic hyperplasia (BPH), prostatitis, infection and DRE increase serum PSA level [15]. In contrast, in 20–40% of patients with prostate cancer that is confined to the prostate itself, the PSA level is less than or equal to 4 ng/ml and does not increase. Therefore, this diagnostic test lonely has a low screening value [16–18]. Needle biopsies are also carried out in advanced stages of the cancer for microscopic examination [19]. Screening tests can identify people who may have a chance of developing the disease before detection and any symptoms [20–23]. Experimental and clinical observations indicate that age, geographical area, ethnic difference, family history, obesity, androgens and various genetic alterations have a role in the pathogenesis of prostate cancer [24–28]. The genetic alterations involved in tumorigenicity include Somatic copy number alterations (SCNAS), structural rearrangement, point mutations, single nucleotide polymorphisms (SNPs) and miRNAs [20]. SNPs are important in genomic studies because there is a significant association between them and various biological traits. This genetic marker is responsible for over 90% of genetic variations that affect the function of genes and cause the difference in the susceptibility of individuals to cancer [29–31]. There are numerous ways for efficient genomic studies, such as multi-stage approach. In this method, a smaller number of people can be used to detect subjects’ genotypes. In the first stage, a complete set of SNPs is examined in a limited number of individuals (according to the normal distribution, individuals are selected from two extremes to make the most difference) at liberal *p*-value. In the next stages, the SNPs selected in the first phase are studied in larger population at more stringent *P*-value. Eventually, from the several candidate SNPs in the first phase, only a small number of SNPs are in actual association with the target trait [32, 33].

SNPs that were investigated in this study are rs2735839, rs721048, rs4242382, rs16901979 and rs1447295. rs4242382, rs16901979 and rs1447295 are located on 8q24 locus. The 8q24 is a susceptibility locus for a wide-range of cancers [23]. This locus, commonly referred to as the gene desert, is highly conserved and consists of three regions (Region 1: 128.54–128.62 Mb; region 2: 128.12–128.28 Mb; region 3: 128.47–128.54 Mb) based on fine mapping [34]. The nearest genes to this chromosomal region are MYC (telomeric end) and FAM84B (centromeric end) [35]. The mechanism by which 8q24 can lead to prostate cancer is still not well understood [23], but there are assumptions that these variants can change the MYC protein expression and affect the Wnt signaling pathway [35]. rs4242382 is located at 230 Kb from MYC gene, and along with rs16901979 are in 8q24 region 2 [23]. rs1447295 C > A is located about 263 kilobases from MYC telomeric end in 8q24 region 1 [34–38]. rs2735839 G/A, an intergenic polymorphism is located on chromosome 19q13.33, at 600 base pairs downstream of the Kallikrein – related peptidase 3 (KLK3) gene untranslatable region [39–41]. The KLK3 gene encodes the prostate-specific antigen, which is widely used in the screening of prostate cancer [42]. rs721048 is located in the EHBP1 gene intron at 2P15. This gene encodes Eps15 homology domain binding protein, which binds clathrin-dependent endocytosis to the cytoskeleton [22].

The purpose of this study was to evaluate the association of rs16901979, rs4242382, rs1447295, rs2735839 and rs721048 with prostate adenocarcinoma and clinical information compared to BPH through multi-stage approach in Iranian population. The published results of various studies in different population are sometimes similar and in some cases different. To date, these polymorphisms have not been studied in Iranian populations. Associated polymorphisms with prostate cancer can be a candidate biomarker for screening test.

## Methods

### Patients

In this case-control study, 103 men with prostate adenocarcinoma (case) and 100 age-matched men with BPH (control) who referred to Shahid Hasheminejad Hospital in Tehran. Iran, from January 2016 to June 2017 were assessed. The sampling was carried out randomly. Diagnosis was made based on the PSA level, DRE, prostate biopsy and physician confirmation so only prostate cancer patients with adenocarcinoma and BPH with no history of cancer were selected in this study. BPH was used as a control because the biopsy showed that the person did not have cancer. Selection of controls derived from the same ethnic population as cases. Patients’ clinical data included age at diagnosis, serum PSA level, prostate volume, Gleason score, extraprostatic extension and perineural invasion.

Written informed consent was obtained from all participants. This research and all methods were performed in accordance with the ethical principles, the national norms, standards, relevant guidelines and regulations for conducting Medical Research in Iran. The study has been approved by the research ethics committee of Kharazmi University (Azizolah Habibi: Organic chemistry, Jafar Jahan Panah: Physics, Mahdi Abbasi Sarmadi: International law, Ali Akbar Imani: Clergy, Mahdi Akbarzadeh: Biostatistics/Epidemiologist, Mahdi Tehranidoost: Biomedical Ethics, Fatemeh Javaheri: Sociology, Jafar Hasani: Clinical Neuropsychology, Alireza Moradi: Clinical Psychology and Zolfaghar Shaker: Communication Sciences) (Approval ID: IR.KHU.REC.1398.002). Peripheral blood samples were taken from patients before initiation of any systemic treatment. The sample size was calculated through the following formula: N = (Z1 – α /2)^2^ p (1-p) / d^2^, Z1 – α /2 = 1.96, *p* = 0.72, d = 0.1, α = 0.05, *N* = 78. According to this calculation, the minimum number of people surveyed in this study was 78 patients, however, more people were studied.

### DNA extraction

Genomic DNA was extracted from peripheral blood lymphocytes using FavorPrep™ Blood Genomic DNA Extraction Mini Kit (Favorgen, Taiwan) instruction. The quality and quantity of each DNA sample were verified using 1.5% agarose gel electrophoresis and NanoDrop™ spectrophotometer respectively. The 260/280 ratio was 1.8.

### SNP genotyping

Tetra-primer amplification refractory mutation system PCR (T-ARMS-PCR) assay was used for SNP genotyping of each participant. The primer design was done using the Primer1 database and validated through the Primer-BLAST-NCBI database. In this method, four primers in a reaction are used for genotyping, which include two non-specific outer primers and two allele-specific inner primers. In order to enhance the specificity, mismatches are introduced at the first and third position from the 3′ end of each of the two inner primers. Primer sequences, the nucleotide determining the alleles of SNP (bold letters) and primer products are shown in the Table 1. Positive control (C+) is the product of two non-specific outer primers. Negative control was also used to ensure the absence of contamination in PCR.

**Table 1:** Primer sequences and primer products

	rs721048	rs4242382	rs16901979	rs1447295	rs2735839
Forward inner	CCCTGAGGATTATCAAGGTCCCTTTG***A***	GAACATTTTGTCCCTCTAGTTATCTTCAC***G***	AGCAGTGTTAATGATTTAGCATTACTTGT***A***	AAGTGCCATTGGGGAGGTATGTAAGA***C***	GCCTAGGGATCTGGTTCTGTCTTGTGGAC***G***
Reverse inner	CTGTTCCACACTGAGGTCCTTATGC***C***	TACTGCCTGATTCTTGATGGGCCGG***T***	TATCTCAAAAATACCATTTGCCAAA***G***	TCCTGTTGCTTTTTTTCCATAGCGC***T***	CCTTCTTGGGATAGCCCCATGGTCCAAT***T***
Forward outer	TCCGAGTACTTCCTCACATAAACCCTGTG	TTCTCTTTTCCATTCCTGTCCTCTCCC	ACATATTTCAGAGGCACATAACTCAA	ATTCTAGAACACATATTGCCTGCCACTGA	CTGTTAGCATGAATCATCTGGCACGGCC
Reverse outer	AGCATGAATTTAGGGTGGCAGAGAGAGA	TTATGAACATCTGCATAGAGGGACGCTG	TATAACAACATTTTGCTCCATCAGTTAC	CTTAAATTTCTTTTCCTCCCAGATTTTCCC	TAACCTCAGGGTGGGAATTCCTCCAGCA
Primer products	**A (mutant)** = 268 bp **G (wild)** = 354 bp **A/G =** 268/354 bp **C+ =** 569 bp	**G (wild)** = 233 bp **A (mutant)** = 355 bp **A/G** = 355/233 bp **C+** = 532 bp	**A (mutant)** = 278 bp **C (wild)** = 323 bp **A/C** = 278/323 bp **C+** = 545 bp	**C (wild)** = 152 bp **A (mutant)** = 228 bp **A/C** = 228/152 bp **C+** = 327 bp	**G (wild)** = 179 bp **A (mutant)** = 268 bp **A/G** = 268/179 bp **C+** = 388 bp

MAF is minor allele frequency Which should be greater than 0.01 to confirm SNP in that genetic region. According to the 1000 genomes project, MAF has been reported in rs721048 (A = 0.0944), rs4242382 (0.1873), rs16901979 (A = 0.2119), rs1447295 (A = 0.1811) and rs2735839 (A = 0.3245).

The PCR reactions were at a final volume of 25 μL and contained 12.5 μL. TAQ DNA POLYMERASE 2X MASTER MIX RED, 0.75 μL of each primer (10 pmol/μl), 2 μL DNA. (50 NG/μl) and 7.5 μL. PCR-GRADE water. Initially using Gradient PCR, the annealing temperature was selected for rs721048, rs4242382, rs16901979 and rs1447295 polymorphisms, 60^°^c, 63^°^c, 57^°^c and 60^°^c respectively. The thermal cycling condition of all polymorphisms includes: 1) PRE-DENATURATION: 95^°^c. 5 min. 1 cycle, 2) DENATURATION: 94^°^c. 30s. 32 cycles, 3) ANNEALING: 30s. 32 cycles. 4)EXTENSION: 72^°^c. 30s. 32 cycles and 5) FINAL EXTENSION: 72^°^c. 3 min. 1 cycle.

### Statistical analysis

Chi-squared and Fisher’s exact test with *p* < 0.3 in the first stage and *p* < 0.05 in the second stage were used to evaluate genotype frequency, allelic frequency, Hardy-Weinberg equilibrium (HWE), multiplicative and additive genetic models. To investigate the SNPs associated with prostate cancer and Gleason score, in the multiplicative and additive models and effect modification, Odds Ratios (OR) with 95% confidence interval (95% CI) were calculated by logistic regression and chi-squared test. Statistical significance was defined as *p* < 0.016 in order to address multiple-test issues by examining 3 SNPs in the second stage and applying a Bonferroni correction. Statistical analyses were performed using SPSS v.25.0.

## Results

### Patients characteristics (stage I)

In the first stage, 59 men were selected which included 30 men with prostate adenocarcinoma and 29 men with BPH. The selection of subjects in the prostate adenocarcinoma group, which included the unhealthiest men, according to Gleason score > 7, positive results of perineural invasion and Extraprostatic extension was done that individuals should have at least two of these criteria. The choice of subjects in the BPH group, which included the healthiest, was according to PSA < 4. In the case and control groups, the age range was between 50–84, 47–78 and the mean age was 71.77 ± 9.22, 62.66 ± 7.848.

### Statistical analysis (stage I)

In the first stage, a complete set of SNPs with liberal *P*-value (< 0.3) were examined to find meaningful polymorphisms. The genotype frequencies of these polymorphisms in each of the two groups are reported separately in the Table 2. There was no significant difference between genotype distribution of rs721048 (*p* = 0.474) and rs16901979 (*p* = 0.612) in case and control groups. With the Chi-square test, prostatic cancer (*P* = 0.952, *P* = 0.612) and BPH (*P* = 0.72, *P* = 0.706) groups in relation to rs721048 and rs16901979 respectively were under HWE. Therefore, multiplicative and additive genetic models were examined to evaluate the association of these polymorphisms and the incidence of cancer. In this study, GG (rs721048) and CC (rs16901979) are wild genotypes, so they are considered as reference genotypes. According to the Table 3 via Fisher’s exact test and logistic regression, none of these two polymorphisms were associated with prostate cancer under the multiplicative and additive genetic models. rs16901979 and rs721048 were not significant in any of the analysis assay, so they were eliminated and in the next stage, only polymorphisms rs4242382, rs2735839 and rs1447295 were evaluated.

**Table 2:** Genotype frequency of polymorphisms in both groups (stage I)

dbSNP		Case n (%)	Control n (%)	*P*-Value
rs721048 (A)	*Genotype*			
	*AA*	0 (0)	2 (6.9)	0.474
	*AG*	7 (23.3)	5 (17.2)	
	*GG*	23 (76.7)	22 (75.9)	
rs4242382 (A)	Genotype			
	AA	1 (3.3)	0 (0)	0.236
	AG	7 (23.3)	3 (10.3)	
	GG	22 (73.3)	26 (89.7)	
rs16901979 (A)	Genotype			
	AA	1 (3.3)	1 (3.4)	0.612
	AC	1 (3.3)	3 (10.3)	
	CC	28 (93.3)	25 (86.2)	
rs1447295 (A)	Genotype			
	AA	0 (0)	0 (0)	0.171
	AC	7 (25.9)	3 (10.3)	
	CC	20 (74.1)	26 (89.7)	
rs2735839 (A)	Genotype			
	AA	2 (6.7)	0 (0)	0.005
	AG	6 (20)	0 (0)	
	GG	22 (73.3)	29 (100)

**Table 3:** Multiplicative and additive genetic models

dbSNP	Multiplicative model		Additive model		
rs721048	A vs G		AA vs GG	AG vs GG	GG
	OR	0.719	1.091	1.339	1 (Reference)
	95%CI	0.249–2.078	0.967–1.231	0.369–4.855	
	*P*-Value	0.541	0.489	0.656	
rs16901979	A vs C		AA vs CC	AC vs CC	CC
	OR	0.558	0.893	0.298	1 (Reference)
	95%CI	0.127–2.449	0.053–15.036	0.029–3.048	
	*P*-Value	0.487	1.000	0.352	

### Patients characteristics (stage II)

At this stage, genotyping of the three selected polymorphisms was performed in the rest of the participants (144 men). In the case (73 men) and control (71 men) groups, the age range was between 48–86, 47–81 and the mean age was 66.09 ± 7.8, 66.21 ± 7.9. All participants of the study (203 men) included 103 prostate adenocarcinomas with age range of 48–86 and 100 BPH with age range of 47–81. The mean age in the case group was 67.75 ± 8.61 and 65.18 ± 8.24 in the control group and totally in the 203 men was 66.48 ± 8.41. The clinical and demographic information of the participants are presented in Table 4.

**Table 4:** The clinical and demographic information of the participants

		Prostate cancer n (%)	BPH n (%)
Age	< 60	17 (16.5)	26 (26)
	60–69	37 (35.9)	41 (41)
	≥70	49 (47.6)	33 (33)
PSA (ng/ml)	≤4	13 (12.6)	19 (19)
	4.1–10	47 (45.6)	56 (56)
	> 10	43 (41.7)	25 (25)
Gleason score	< 7	14 (13.6)	–
	7	47 (45.6)	–
	≥8	42 (40.8)	–
Perineural invasion	+	65 (63.1)	–
	–	38 (36.9)	–
Extra prostatic extension	+	18 (17.5)	–
	–	85 (82.5)	–

### Statistical analysis (stage II)

Since the statistical analysis of the first stage was performed only for the selection of meaningful polymorphisms in order to examination in the second stage, the statistical analysis of this stage included all participants (203 men). First, in three polymorphisms (rs4242382, rs1447295, rs2735839), the Hardy-Weinberg equilibrium was studied in two groups by using Chi-square test. There was no statistically significant difference between the observed and expected genotype frequency in the adenocarcinoma (rs4242382 [*p* = 1], rs1447295 [*p* = 0.854], rs2735839 [*p* = 0.655]) and BPH (rs4242382 [p = 1], rs1447295 [p = 1], rs2735839 [*p* = 1]) groups. The genotype frequency of these polymorphisms is reported in the Table 5.

**Table 5:** Genotype frequency of polymorphisms, multiplicative and additive genetic models

dbSNP		Case n (%)	Control n (%)	*P*-Value	Multiplicative model		Additive model		
rs4242382	Genotype				A vs G		AA vs GG	AG vs GG	GG
	AA	1 (1)	0 (0)	0.001	OR	4.466	3.56	4.561	1(Reference)
	AG	23 (22.3)	6 (6)		95%CI	1.791–11.136	0.14–88.76	1.769–11.758	
	GG	79 (76.7)	94 (94)		*P*-Value	0.001	0.438	0.001	
rs1447295	Genotype				A vs C		AC vs CC	CC	
	AA	0 (0)	0 (0)	0.005	OR	3.432	3.721	1 (Reference)	
	AC	19 (19.2)	6 (6)		95%CI	1.341–8.786	1.418–9.767		
	CC	80 (80.8)	94 (94)		*P*-Value	0.007	0.005		
rs2735839	Genotype				A vs G		AA vs GG	AG vs GG	GG
	AA	3 (2.9)	1 (1)	0.00	OR	3.927	4.424	5.038	1 (Reference)
	AG	41 (39.8)	12 (12)		95%CI	2.085–7.397	0.44–43.56	2.44–10.38	
	GG	59 (57.3)	87 (87)		*P*-Value	0.00	0.307	0.00	

The association of rs4242382 (*P* = 0.001, OR [95% CI] =4.466 [1.791–11.136]), rs1447295 (*P* = 0.007, OR [95% CI] = 3.432 [1.341–8.786]) and rs2735839 (*P* = 0.00, OR [95% CI] =3.927 [2.085–7.397] with prostate adenocarcinoma was observed under the multiplicative genetic model. In this study, GG (rs4242382, rs2735839) and CC (rs1447295) are wild genotypes, so they are considered as reference genotypes. According to the additive genetic model, AG (rs4242382) is associated with prostate cancer (OR = 95% CI) = 4.561 [1.769–11.758], *P* = 0.00) using GG as a reference genotype. There was a significant difference between genotype frequency AG + AA in the case compared to control group (OR [95% CI] = 4.759 [1.853–12.224], *P* = 0.00). AC (rs1447295) is associated with prostate cancer (OR [95% CI] = 3.721 [1.418–9.767], *P* = 0.005) using CC as a reference genotype. AG (rs2735839) is associated with prostate cancer (OR = 95% CI) =5.038 [2.44–10.38], *P* = 0.00) using GG as a reference genotype. There was a significant difference between genotype frequency AG + AA in the case compared to control group (OR [95% CI] = 4.991 [2.475–10.065], *P* = 0.00). All of these associations remained significant after Bonferroni correction (*p* < 0.016).

In Table 6, genotype and allelic frequency, the OR[95%CI] (logistic regression) and *P*-value (Fisher’s exact test) of risk allele compared to wild allele at three categories of Gleason score were presented. According to this table, there is a significant difference between the frequency of allele A vs G in all three categories of Gleason score at rs42423820 and rs2735839. At rs1447295, there is a significant difference between frequency of allele A vs C only in the Gleason score ≥ 8. After Bonferroni correction, rs4242382 did not associate with Gleason Score = 7 and rs1447295 with any Gleason Score categories.

**Table 6:** Genotype and allelic frequency, the OR[95%CI] and *P*-value of risk allele compared to wild allele at three categories of Gleason score

rs4242382		AA n (%)	AG n (%)	GG n (%)	A	G	OR (95%CI)	*P*-Value
Control		0 (0)	6 (6)	94 (94)	6 (0.03)	194 (0.97)	1(reference)	–
Gleason score	< 7	0 (0)	5 (35.7)	9 (64.3)	5 (0.178)	23 (0.821)	7.029 (1.988–24.856)	0.005
	7	0 (0)	8 (17)	39 (83)	8 (0.08)	86 (0.91)	3.008 (1.013–8.933)	0.039
	≥8	1 (2.4)	10 (23.8)	31 (73.8)	12 (0.14)	72 (0.85)	5.389 (1.95–14.894)	0.00
rs2735839		**AA**	**AG**	**GG**	**A**	**G**	**OR (95%CI)**	***P*-Value**
Control		1 (1)	12 (12)	87 (87)	14 (0.07)	186 (0.93)	1(reference)	–
Gleason score	< 7	0 (0)	7 (50)	7 (50)	7 (0.25)	21 (0.75)	4.429 (1.608–12.199)	0.007
	7	1 (2.1)	15 (31.9)	31 (66)	17 (0.18)	77 (0.81)	2.933 (1.378–6.244)	0.004
	≥8	2 (4.8)	19 (45.2)	21 (50)	23 (0.27)	61 (0.72)	5.009 (2.427–10.339)	0.00
rs1447295		**AA**	**AC**	**CC**	**A**	**C**	**OR (95%CI)**	***P*-Value**
Control		0 (0)	6 (6)	94 (94)	6 (0.03)	194 (0.97)	1(reference)	–
Gleason score	< 7	0 (0)	3 (21.4)	11 (78.6)	3 (0.107)	25 (0.89)	3.88 (0.913–16.494)	0.084
	7	0 (0)	8 (17)	39 (83)	8 (0.08)	86 (0.91)	3.008 (1–8.933)	0.073
	≥8	0 (0)	8 (21)	30 (78.9)	8 (0.105)	68 (0.89)	3.804 (1.274–11.359)	0.026

Using Fisher’s exact test, no significant difference was found between the distribution of genotype at rs4242382, rs1447295 and rs2735839 with the perineural invasion and extraprostatic extension (Table 7).

**Table 7:** Genotype distribution of polymorphisms and *p*-value in mentioned clinical features

rs4242382		AA	AG	GG	*P*-Value
Perineural invasion	+	1 (1.5)	13 (20)	51 (78.5)	0.763
	–	0 (0)	10 (26.13)	28 (73.7)	
Extraprostatic extension	+	1 (5.6)	4 (22.2)	13 (72.2)	0.27
	–	0 (0)	19 (22.4)	66 (77.6)	
rs1447295		**AA**	**AC**	**CC**	***P*-Value**
Perineural invasion	+	0 (0)	11 (17.7)	51 (82.3)	0.635
	–	0 (0)	8 (21.6)	29 (78.4)	
Extraprostatic extension	+	0 (0)	4 (23.5)	13 (76.5)	0.735
	–	0 (0)	15 (18.3)	67 (81.7)	
rs2735839		AA	AG	GG	***P*-Value**
Perineural invasion	+	3 (4.6)	25 (38.5)	37 (56.9)	0.600
	–	0 (0)	16 (42.1)	22 (57.9)	
Extraprostatic extension	+	1 (5.6)	6 (33.3)	11 (61.1)	0.527
	–	2 (2.4)	35 (41.2)	48 (56.5)	

### Effect modification

In this study, age and PSA were investigated as an effect modification. According to Table 8, Age and PSA were categorized into 3 subgroups. The association of rs4242382, rs2735839 and rs1447295 with prostate cancer was investigated in each subgroup (df = 2). However, in none of these polymorphisms, total OR was significantly different with odds ratio of subgroup, so age and PSA are not considered as effect modification in this study.

**Table 8:** Effect modification

		Age				PSA			
dbSNP		< 60	60–69	≥70	Total	≤4	4.1–10	> 10	Total
rs4242382	OR	2.41	6.98	4.04	4.46	1.8	9.29	4.66	4.46
	X^2^	0.756				1.84			
rs2735839	OR	15.69	4.24	2.22	3.92	–	5.73	1.62	3.92
	X^2^	3.303				4.68			
rs1447295	OR	0.86	3.76	6.72	3.43	4.82	3.08	2.57	3.43
	X^2^	1.848				0.184			

## Discussion

In the present study, polymorphisms rs16901979, rs1447295 and rs4242382, rs2735839 and rs721048 were evaluated in the prostate cancer compared to control group (BPH) in Iranian population for the first time. In the first stage [43], which included the unhealthiest and healthiest participants, rs16901979 and rs721048 did not show any significant association with prostate adenocarcinoma. There was no significant difference between the genotype frequency of rs16901979 (*P* = 0.671) and rs721048 (*P* = 0.474) in both case and control groups. According to the multiplicative and additive genetic models, the association of these polymorphisms with prostate adenocarcinoma was not observed, so rs16901979 and rs721048 were eliminated at first stage. There was a significant difference between genotype distribution of rs4242382, rs1447295 and rs2735839 in both case and control groups in the first stage (*P* = 0.236, 0.171, 0.005). Therefore, these polymorphisms were chosen for the second stage studies as candidate SNPs. According to all participants (203 men), rs4242382 (AG Vs GG) (OR [95% CI] = 4.561 [1.769–11.758], P = 0.00), rs1447295 (AC Vs CC) (OR [95% CI] = 3.721 [1.418–9.767], *P* = 0.005) and rs2735839 (AG Vs GG) (OR [95% CI] = 5.038 [2.444–10.384], *P* = 0.0) were associated with cancer under additive genetic model. Also, according to the multiplicative genetic model, the association of these three polymorphisms, rs4242382 (A vs G) (OR [95% CI] = 4.466 [1.791–11.136] *P*-Value = 0.001), rs1447295 (A vs C) (OR [95% CI] = 3.432 [1.341–8.786] *P*-Value = 0.007) and rs2735839 (A vs G) (OR [95% CI] = 3.927 [2.085–7.397] *P*-Value = 0.00) with prostate adenocarcinoma was reported. All of these associations remained significant after Bonferroni correction (*p* < 0.016). There was a significant difference between the allelic frequency of A vs G (rs4242382, rs2735839) at all three classes of Gleason score and A vs C (rs1447295) at Gleason score ≥ 8 Which probably indicates the association of this polymorphism (rs1447295) with the invasive prostate cancer but after Bonferroni correction, rs4242382 did not associate with Gleason Score = 7 and rs1447295 with any Gleason Score categories. No significant difference was found between genotype frequency and clinical features such as perineural invasion and extraprostatic extension.

Among genomic changes that are associated with the development of prostate cancer, the variants in 8q24 chromosomal region are very important. In European and American populations, several variants have been identified in this locus for prostate cancer, but similar studies have been conducted less in Asian populations [23]. More than 11 Genome Wide Association Study (GWAS) have identified strong association between the 8q24 chromosome and the risk of this cancer. Variants of this locus can affect the regulation or transcription of a causative gene outside this region. MYC (proto-oncogene) at the telomeric end is a strong candidate gene in this field. The transcription factor encoded by c-MYC regulates the expression of multiple genes responsible for cell proliferation, cell differentiation and apoptosis [35]. FAM84B (at the centromeric end) expression, increases during prostate tumorigenesis and progression [35, 37]. rs2735839 G/A is located at 600 base pairs downstream of the KLK3 gene (PSA) 3′ untranslated region, which codes PSA [40, 41]. PSA (30-kDa) is a protease, which is released by the prostate gland epithelial cells into seminal fluid and blood. PSA can cleave a number of proteins, such as the insulin-like growth factor binding protein (IGFBP2,3,5), Parathyroid hormone-related protein (PTH-related protein), latent TGF-β2, fibronectin and laminin (extracellular matrix components) Whose destruction is the first step in tumorigenesis, metastasis and development of prostate cancer [44–46]. In prostate cancer, serum PSA level usually increases, which can induce mutations in P53 and up-regulation of the B-cell lymphoma 2 protein that inhibits apoptosis in tumor cells [47]. rs721048 A/G is located in the intron of EHBP1 gene. This gene encodes Eps15 homology domain binding protein. Upregulation of the NPF motif contained in EHBP1 causes degradation of the actin structure. However, the precise mechanism of EHBP1 in the onset and progression of prostate cancer are still unknown [22].

The effect modification is seen when the association of exposure and outcome varies according to a third factor, Indeed, the exposure can have a different effect among the different subgroups [48, 49]. We investigated whether age and PSA were an effect modifier in association between exposure (rs4242382, rs2735839 and rs1447295) and outcome (prostate cancer), but the effect of rs4242382 (X^2^ = 0.756, X^2^ = 1.84), rs2735839 (X^2^ = 3.303, X^2^ = 4.68) and rs1447295 (X^2^ = 1.848, X^2^ = 0.184) on prostate cancer was not modified by age and PSA respectively. Therefore, exposure effect is homogeneous between different ages and serum PSA level categories.

8q24 was initially identified by genome-wide linkage study in the Icelandic families’ genome. Follow up these subjects, displayed that the microsatellite DG8S737 (repeat of the two nucleotides AC) and rs1447295 had the highest association with the risk of prostate cancer [50]. During a cohort study by Suuriniemi et al., rs1447295 had strong association with prostate cancer in 597 prostate cancer patients and 548 European-American controls [51] which was similar to the results of this study. The results of this study for rs4242382 follow the Zheng et al*,* studies which observed 1.3 to 1.9 times Increased the risk of prostate cancer among European men with either one or both rs4242382 risk alleles (A) (OR = 2.90; 95% CIs = 1.02–8.25 and OR = 2.90; 95% CIs = 1.02–8.25) [52]. The effect of rs16901979 has been reported in African-American population more than the Asian ethnic [23]. During a study on African ethnic related to rs16901979, genotype AA and allele A were reported with an increased risk of prostate cancer (OR = 1.84 [95%CI] = 1.26–2.69, *P* = 0.002 and OR = 1.36, 95% CI = 1.13–1.64, *P* = 0.001) as well as association of AC + AA with Gleason score ≥ 7 (invasive prostate cancer) [53, 54] which is contrary to the present study due to racial differences. In a study by Cheng-Xiao Zhao et al., The results indicate that 8q24 rs4242382-A may be related to the increased sensitivity to prostate cancer in Asian, Caucasian, and African American populations. Therefore, this polymorphism can be a multi-ethnic marker [23]. During a meta-analysis, a total of 20,239 cases and 20,439 controls were studied for rs1447295 C > A and 1850 cases and 2090 controls for rs16901979 C > A. rs1447295 was associated with the risk of prostate cancer in the Caucasian and Asian, but not African-American. The effect of rs16901979 was high among African-American more than the Asian [34].

In the first GWAS by Eles and colleagues, rs2735839 was identified as a risk factor for prostate cancer [55] Which showed a stronger association with the PSA level than previous polymorphisms [56]. Pomerantz et al., were found that rs2735839 (A) has been related to the mortality in prostate cancer [57]. However, during a meta-analysis between three KLK3 polymorphisms (2018), no association was found about rs2735839 and prostate cancer [58].

rs721048 (A) was first recognized by the GWA studies in the Caucasian population as a risk factor for prostate cancer. Through a meta-analysis, which included 48,135 cases and 10,254 controls, Xiang Ao et al., showed strong association of this polymorphism with prostate cancer (OR = 1.14, 95% CI = 1.11–1.17, *P* = 0.000) [22]. Unlike the Icelandic population, this polymorphism is not associated with cancer in the Netherland, Spain and Sweden population [43] as well as present study.

## Conclusions

The results of this study indicate that polymorphisms rs2735839, rs4242382 and rs1447295 are associated with prostate cancer predisposition in Iranian population therefore, they may be considered as a biomarker for prostate cancer. Exposure effect is homogeneous between different ages and serum PSA level categories. Association of allele A (rs4242382, rs2735839) with all three Gleason score categories and rs1447295 (A) only at Gleason score ≥ 8, was observed. In future studies, it is suggested that these confirmed polymorphisms be examined in a larger population and separately in each of the Iranian ethnic.

### Research limitations

- Generally, markers should be validated by testing their effectiveness in determining the target phenotype in independent population and different genetic backgrounds, which is referred to as marker validation. In the present study, the association was studied totally in Iranian population. Therefore, each of these polymorphic markers should be investigated separately in each Iranian race.
- Due to the limited population of the present study, the association of these polymorphisms in a larger population should be investigated for a more accurate conclusion.
- Performing NGS on patient samples can provide more comprehensive results regarding this cancer in the Iranian population.

## Data Availability

All data generated or analyzed during this study are included in this published article. Details (Phenotype and genotypes in individuals for each of the five polymorphisms) are available from the corresponding author, on reasonable request.

## Abbreviations

ASIR: Age-Standardized Incidence Rate
ASMR: Age-Standardized Mortality Rate
PSA: Prostate specific antigen
DRE: Digital rectal exam
BPH: Benign prostatic hyperplasia
SCNAS: Somatic copy number alterations
SNPs: Single nucleotide polymorphisms
KLK3: Kallikrein – related peptidase 3
T-ARMS-PCR: Tetra-primer amplification refractory mutation system PCR
HWE: Hardy-Weinberg equilibrium
GWAS: Genome Wide Association Study
IGFBP2,3,5: Insulin-like growth factor binding protein
PTH-related protein: Parathyroid hormone-related protein
PCR: Polymerase chain reaction
